# The effect of lifestyle on the mortality associated with respiratory diseases in the general population

**DOI:** 10.1038/s41598-023-34929-8

**Published:** 2023-05-22

**Authors:** Hiroaki Murano, Sumito Inoue, Kento Sato, Masamichi Sato, Akira Igarashi, Shouichi Fujimoto, Kunitoshi Iseki, Toshiki Moriyama, Yugo Shibagaki, Masato Kasahara, Ichiei Narita, Kunihiro Yamagata, Kazuhiko Tsuruya, Masahide Kondo, Koichi Asahi, Tsuyoshi Watanabe, Tsuneo Konta, Masafumi Watanabe

**Affiliations:** 1 Department of Cardiology, Pulmonology, and Nephrology, Yamagata University Faculty of Medicine, Yamagata, Japan; 2 Steering Committee of Research on Design of the Comprehensive Health Care System for Chronic Kidney Disease (CKD) Based on the Individual Risk Assessment by Specific Health Check, Fukushima, Japan

**Keywords:** Epidemiology, Risk factors, Prognosis, Public health

## Abstract

Lifestyle factors, including smoking habit, diet, and physical activity, affect the prognosis of various diseases. We elucidated the effect of lifestyle factors and health status on deaths from respiratory diseases in the general Japanese population using data from a community health examination database. Data of the nationwide screening program of the Specific Health Check-up and Guidance System (Tokutei-Kenshin), targeting the general population in Japan, from 2008 to 2010 were analyzed. The underlying causes of death were coded according to the International Classification of Diseases (ICD)-10. The hazard ratios of the incidence of mortality associated with respiratory disease were estimated using the Cox regression model. This study included 664,926 participants aged 40–74 years, who were followed up for 7 years. There were 8051 deaths, including 1263 (15.69%) deaths from respiratory diseases. The independent risk factors of mortality associated with respiratory diseases were male sex, older age, low body mass index, no exercise habit, slow walking speed, no drinking habit, smoking history, history of cerebrovascular diseases, high hemoglobin A1c and uric acid levels, low low-density lipoprotein cholesterol level, and proteinuria. Aging and decline of physical activity are significant risk factors for mortality associated with respiratory diseases, regardless of the smoking status.

## Introduction

Japanese people are known to have the longest average life expectancy worldwide^1^. As a result, the number of elderly people in Japan is increasing, and the etiology of disease is changing. Currently, approximately 30% of the Japanese population reportedly dies from malignant neoplasm, followed by heart disease, senility, cerebrovascular disease, and pneumonia^2^. Regarding the causes of death by organ, approximately 20% of deaths from malignant neoplasm are due to lung cancer, which is the most common cause of malignant neoplasm-related deaths. Deaths due to pneumonia and aspiration pneumonia are the fifth and sixth leading causes of deaths in Japan. We previously conducted a follow-up study of > 3000 residents of Takahata town, Yamagata Prefecture, Japan, who had undergone health checkups. The analysis of the causes of death after 7 years revealed that deaths due to respiratory diseases, including lung cancer, were the most common, accounting for approximately 30% of all deaths^3^. Elderly admitted to hospitals due to a disease condition require high treatment cost and long hospitalization^4^. Even if the disease causing hospitalization improves as a result of treatment, the patient may not be able to leave the hospital or may require long-term rehabilitation due to the decline in the activity of daily living (ADL). Furthermore, it may also cause a decline in cognitive function^5–7^. In Japan, the concept of “healthy life expectancy” exists. Despite the fact that the life expectancy of both men and women has been extended, approximately 10 years of their lives will require nursing care. Thus, the loss of a healthy life expectancy is a serious problem^8^.

Lifestyle is well known to influence the onset and prognosis of various diseases. Chronic obstructive pulmonary disease (COPD) is among the lifestyle-related diseases of the respiratory system, with smoking habits playing a major role in its pathogenesis. Physical activity plays a more significant role in the prognosis of patients with COPD than the decline of pulmonary function^9^. A previous study on individuals who had undergone a physical examination showed that regularly walking reduced the risk of death from pneumonia^10–14^.

Habitual exercise influences the progression and prognosis of heart disease, diabetes, and dyslipidemia^15^. However, there have been few reports on the impact of lifestyle on the prognosis of various respiratory diseases in the general population who had undergone health checkups.

Therefore, we aimed to clarify the effects of lifestyle and medical conditions on the health status of the general population using data obtained from the Community Health Examination System.

## Results

### Profiles of participants

Table 1 shows the demographic profiles of the part included in this study. Among the 664,926 participants, 42.76% were men and the mean age was 62.3 years (39–75 years). Moreover, 31.70% of the studied individuals exercised for ≥ 30 min, and 38.17% regularly walked or engaged in physical activity. Furthermore, 38.26% of the participants reported walking faster as compared to the other participants of the same sex and age. Altogether, 15.56% and 46.50% of the participants had a smoking history and drinking habits, respectively. The history of cerebrovascular disease, cardiovascular disease, chronic renal failure, or anemia was present in 3.49%, 5.40%, 0.77%, and 8.65% of the participants, respectively.

**Table 1:** Profiles and laboratory data of participants.

Profiles	
Sex (men)	284,320 (42.76)
Age (year)	62.3 ± 8.8
Height (cm)	157.6 ± 8.7
Body weight (kg)	58.4 ± 11.0
Body mass index (kg/m^2^)	23.4 ± 3.5
Waist circumference (cm)	84.2 ± 9.4
Blood pressure (systolic) (mmHg)	129 ± 18
Blood pressure (diastolic) (mmHg)	76 ± 11
Weight gain more than 10 kg since age 20	186,105 (27.99)
Exercise habit for more than 30 min	210,785 (31.70)
Habit of walking and/or physical activity	253,798 (38.17)
Fast walking speed	254,408 (38.26)
Smoking history	103,431 (15.56)
Drinking habits	292,060 (46.50)
Adequate sleep	390,335 (58.70)
Past history	
Cerebrovascular diseases	23,206 (3.49)
Cardiovascular diseases	35,895 (5.40)
Chronic renal failure	5128 (0.77)
Anemia	57,525 (8.65)
Medication	
Hypertention	197,565 (29.71)
Diabetes	36,763 (5.53)
Dyslipidemia	98,828 (14.86)
Laboratory data	
Glucose (mg/dl)	98 ± 22
Hemoglobin A1c (%)	5.7 ± 0.8
Triglyceride (mg/dl)	125 ± 89
High density lipoprotein cholesterol (mg/dl)	61 ± 16
Low density lipoprotein cholesterol (mg/dl)	125 ± 31
Aspartate aminotransferase (IU/l)	25 ± 13
Alanine aminotransferase (IU/l)	22 ± 16
Hemoglobin (g/dl)	13.6 ± 1.5
Hematocrit (%)	41.2 ± 4.1
Red blood cell (× 10^4^/μL)	439 ± 60
Uric acid (mg/dl)	5.3 ± 1.4
Creatinine (mg/dl)	0.73 ± 0.30
Estimated glemerular filtration rate (mL/min/1.73m^2^)	75.9 ± 17.3
Urinary protein (positive)	87,439 (13.15)

Date are expressed as mean ± standard deviation, or number (%).

### Classification of causes of death

All participants were followed up for 7 years; 8051 of them died. The causes of their deaths are summarized in Table 2. Malignant neoplasm was the most common cause of death, accounting for 4159 deaths (62.78% were men) and more than half of all deaths in both sexes. The second most common cause of death was circulatory system disease with 1616 deaths (64.60% were men), accounting for approximately 20% of all deaths in both sexes. The third and fourth most common causes of death was injury, poisoning, or other external causes (920 deaths, 64.46% were men) and respiratory disease (437, 73.23% were men), respectively. Since deaths from malignant neoplasms include deaths related to respiratory disease, we extracted data of 826 deaths from malignant neoplasms of the bronchus and lungs [71.67% (n = 592) were men]. As a result, the total number of deaths from malignant and non-malignant neoplasms of the respiratory system was 1263 (72.21% [n = 912] were men), accounting for 15.69% of all deaths (17.71% and 12.10% of all deaths in men and women, respectively).

**Table 2:** Classification of causes of death.

	Men	%	Women	%	Total	%
Malignant neoplasm	2611	50.69	1548	53.38	4159	51.66
Heart diseases	1044	20.27	572	19.72	1616	20.07
Injury, poisoning, other external causes	593	11.51	327	11.28	920	11.43
Respiratory diseases	320	6.21	117	4.03	437	5.43
Gastrointestinal diseases	193	3.75	79	2.72	272	3.38
Infectious diseases	78	1.51	63	2.17	141	1.75
Neurological diseases	71	1.38	50	1.72	121	1.50
Metabolic endocrine diseases	48	0.93	16	0.55	64	0.79
Musculoskeletal diseases	16	0.31	36	1.24	52	0.65
Genital diseases	32	0.62	12	0.41	44	0.55
Hematological and immunological diseases	17	0.33	9	0.31	26	0.32
Psychiatric diseases	12	0.23	4	0.14	16	0.20
Congenital disease	2	0.04	2	0.07	4	0.05
Skin diseases	1	0.02	3	0.10	4	0.05
Perinatal problems	0	0.00	1	0.03	1	0.01
Unclassifiable diseases	86	1.67	45	1.55	131	1.63
Unknown	27	0.52	16	0.55	43	0.53
	5151		2900		8051	

### Risk of deaths due to respiratory diseases

The causes of deaths from respiratory diseases, including malignant neoplasms, are shown in Table 3. Of the 1263 deaths, the most common cause was malignant neoplasm (592 were men, 64.91%; 234 were women, 66.67%), followed by respiratory infections caused by viruses and bacteria (151 were men, 16.56%; 51 were women, 14.53%). Interstitial pulmonary disease was the third most common cause, followed by obstructive pulmonary disease.

**Table 3:** Deaths due to respiratory diseases including malignant neoplasms.

	Men	%	Women	%	Total	%
Malignant neoplasm	592	64.91	234	66.67	826	65.40
Viral or bacterial infections	151	16.56	51	14.53	202	15.99
Interstitial pulmonary diseases	89	9.76	37	10.54	126	9.98
Obstructive pulmonary diseases	34	3.73	8	2.28	42	3.33
Aspiration	24	2.63	6	1.71	30	2.38
ARDS/pulmonary edema	3	0.33	6	1.71	9	0.71
Respiratory failures	5	0.55	4	1.14	9	0.71
Pneumoconiosis	6	0.66	0	0.00	6	0.48
Lung abscess/pyothorax	4	0.44	1	0.28	5	0.40
Pleural diseases	2	0.22	1	0.28	3	0.24
Others	2	0.22	3	0.85	5	0.40
	912		351		1263	

*ARDS* acute respiratory distress syndrome.

Table 4 shows the results of the analysis for the risk of death from respiratory disease with or without malignant neoplasm in the respiratory tract. Death from respiratory disease, including malignant neoplasm, was significantly associated with sex, older age, lower body mass index (BMI), increased systolic blood pressure, no walking habit or physical activity, slow walking speed, smoking history, drinking habit, adequate sleep, history of cerebrovascular or cardiovascular disease, receiving treatment for hypertension or diabetes, increased levels of fasting blood glucose, hemoglobin A1c (HbA1c), aspartate aminotransferase (AST), UA, and creatinine (Cre), decreased level of high-density lipoprotein cholesterol (HDL-C), low-density lipoprotein cholesterol (LDL-C), and alanine aminotransferase (ALT), and proteinuria.

**Table 4:** Univarite analysis of risk factors in respiratory disease mortality.

	Respiratory diseases including malignancy (n = 1263)				Respiratory diseases excluding malignancy (n = 437)				Malignant neoplasm of respiratory tract (n = 826)			
	Hazard ratio	95% CI		p value	Hazard ratio	95% CI		p value	Hazard ratio	95% CI		p value
Sex (men)	3.595	3.182	4.071	< 0.0001*	3.780	3.069	4.689	< 0.0001*	3.503	3.016	4.082	< 0.0001*
Age (+ 1 year)	1.106	1.095	1.118	< 0.0001*	1.132	1.111	1.155	< 0.0001*	1.094	1.081	1.108	< 0.0001*
Body mass index (+ 1 kg/m^2^)	0.959	0.943	0.976	< 0.0001*	0.902	0.874	0.930	< 0.0001*	0.989	0.968	1.009	0.2727
Waist circumference (+ 1 cm)	0.999	0.993	1.005	0.8277	0.983	0.973	0.993	0.0011*	1.008	1.000	1.015	0.0385*
Systolic blood pressure (+ 1 mmHg)	1.006	1.003	1.009	0.0004*	1.002	0.997	1.008	0.3949	1.007	1.004	1.011	0.0002*
Diastolic blood pressure (+ 1 mmHg)	0.997	0.991	1.002	0.1773	0.986	0.978	0.995	0.0021*	1.002	0.996	1.008	0.5788
Weight gain more than 10 kg since age 20	0.927	0.813	1.055	0.2521	0.744	0.589	0.932	0.0097*	1.039	0.884	1.217	0.6427
Exercise habit for more than 30 min	0.896	0.789	1.016	0.0878	0.663	0.534	0.834	0.0003*	1.042	0.892	1.217	0.6004
Habit of walking and/or physical activity	0.868	0.767	0.982	0.0241*	0.718	0.581	0.886	0.0019*	0.960	0.824	1.119	0.6015
Fast walking speed	0.547	0.480	0.622	< 0.0001*	0.308	0.239	0.391	< 0.0001*	0.719	0.614	0.839	< 0.0001*
Smoking history	2.278	2.016	2.570	< 0.0001*	1.265	0.987	1.601	0.0629	2.947	2.550	3.398	< 0.0001*
Drinking habits	1.142	1.020	1.278	0.0217*	0.876	0.721	1.062	0.1768	1.314	1.142	1.513	0.0001*
Adequate sleep	1.366	1.168	1.606	< 0.0001*	1.124	0.879	1.456	0.3567	1.536	1.256	1.896	< 0.0001*
History of cerebrovascular diseases	2.647	2.148	3.225	< 0.0001*	3.335	2.407	4.504	< 0.0001*	2.289	1.733	2.963	< 0.0001*
History of cardiovascular diseases	2.217	1.845	2.641	< 0.0001*	2.632	1.961	3.462	< 0.0001*	2.000	1.575	2.504	< 0.0001*
History of chronic renal failure	1.313	0.721	2.169	0.3496	2.341	1.064	4.394	0.0361*	0.771	0.276	1.664	0.5455
History of anemia	0.979	0.801	1.184	0.8288	1.096	0.788	1.484	0.5754	0.917	0.709	1.166	0.4893
Medication of hypertension	1.555	1.386	1.741	< 0.0001*	1.516	1.246	1.839	< 0.0001*	1.575	1.367	1.812	< 0.0001*
Medication of diabetes	2.220	1.843	2.650	< 0.0001*	3.435	2.621	4.430	< 0.0001*	1.627	1.248	2.081	0.0005*
Medication of dyslipidemia	0.970	0.825	1.135	0.7098	0.898	0.673	1.175	0.4414	1.009	0.827	1.221	0.9247
Fasting blood glucose (+ 1 mg/dl)	1.009	1.007	1.010	< 0.0001*	1.009	1.006	1.012	< 0.0001*	1.008	1.006	1.010	< 0.0001*
Hemoglobin A1c (+ 1%)	1.268	1.203	1.330	< 0.0001*	1.316	1.211	1.416	< 0.0001*	1.239	1.157	1.319	< 0.0001*
Triglyceride (+ 1 mg/dl)	1.000	1.000	1.001	0.1650	0.999	0.997	1.000	0.0431*	1.001	1.000	1.002	0.0038*
High density lipoprotein cholesterol (+ 1 mg/dl)	0.979	0.975	0.983	< 0.0001*	0.982	0.975	1.018	< 0.0001*	0.978	0.973	0.983	< 0.0001*
Low density lipoprotein cholesterol (+ 1 mg/dl)	0.990	0.988	0.991	< 0.0001*	0.987	0.984	0.990	< 0.0001*	0.991	0.989	0.993	< 0.0001*
Aspartate aminotransferase (+ 1 U/L)	1.004	1.002	1.006	0.0002*	1.005	1.002	1.007	0.0018*	1.004	1.001	1.006	0.0271*
Alanine aminotransferase (+ 1 U/L)	0.996	0.991	0.999	0.0443*	0.996	0.989	1.004	0.2793	0.996	0.990	1.001	0.0889
Hemoglobin (+ 1 g/dl)	1.045	0.983	1.111	0.1578	0.931	0.837	1.038	0.1940	1.104	1.024	1.186	0.0097*
Hematocrit (1%)	1.015	0.993	1.037	0.1883	0.976	0.940	1.014	0.2168	1.032	1.006	1.059	0.0157*
Red blood cell (+ 1 /μL)	0.999	0.998	1.001	0.0588	0.998	0.996	1.002	0.0799	0.999	0.998	1.001	0.2835
Uric acid (+ 1 mg/dl)	1.072	1.055	1.084	< 0.0001*	1.071	1.041	1.090	0.0002*	1.072	1.051	1.086	< 0.0001*
Creatinine (+ 1 mg/dl)	1.334	1.251	1.404	< 0.0001*	1.388	1.269	1.482	< 0.0001*	1.291	1.166	1.389	< 0.0001*
Estimated glomerular filtration rate (+ 1 mL/min/1.73m^2^)	1.000	0.997	1.004	0.9332	1.005	1.000	1.007	0.0427*	0.996	0.992	1.001	0.1067
Urinary protein (positive)	1.789	1.554	2.051	< 0.0001*	2.310	1.845	2.867	< 0.0001*	1.538	1.280	1.834	< 0.0001*

Asterisk means p < 0.05, statistically significant.

Deaths from respiratory disease excluding malignant neoplasms were significantly associated with sex, older age, lower BMI, decreased waist circumference, lower diastolic blood pressure, no weight gain of > 10 kg compared to the weight at 20 years of age, no exercise habit of > 30 min, no walking habit or physical activity, slow walking speed, history of cerebrovascular disease, cardiovascular disease, or chronic renal failure, receiving treatment for hypertension or diabetes, increased fasting blood glucose, HbA1c, AST, UA, and Cre levels, increased estimated glomerular filtration rate (eGFR), lower triglyceride (TG), HDL-C, and LDL-C levels, and proteinuria.

Deaths from malignant neoplasms in the respiratory tract were significantly associated with sex, aging, increased waist circumference, higher systolic blood pressure, slow walking speed, smoking history, drinking habits, adequate sleep, history of cerebrovascular or cardiovascular disease, treatment for hypertension or diabetes, increased fasting blood glucose, HbA1c, TG, AST, hemoglobin, hematocrit, UA, and creatinine levels, lower HDL-C and LDL-C levels, and proteinuria.

The independent risk factors of death from respiratory disease with or without a malignant neoplasm in the respiratory tract was examined through a Cox proportional hazards analysis (Table 5). For deaths from respiratory disease, including malignant neoplasm, the independent risk factors were male sex, older age, lower BMI, no walking habit or physical activity, slow walking speed, smoking history, no drinking habit, history of cerebrovascular disease, increased HbA1c and UA levels, decreased LDL-C level, and proteinuria. For deaths from respiratory disease, excluding malignant neoplasm, we excluded the factor “Waist circumference” from the Cox proportional hazards analysis even though it was significant in the univariate analysis. Because waist circumference was considered to be strongly correlated with BMI. Additionally, we did not include the factor “Habit of walking and/or physical activity” in this analysis. In univariate analyses, “Exercise habit for more than 30 min” and “Habit of walking and/or physical activity” were significant risk factors for death from respiratory disease excluding malignant neoplasm, but as these factors were similar, we included only “Exercise habit for more than 30 min” in the Cox proportional hazards analysis. The independent risk factors were male sex, older age, lower BMI, exercise habit for less than 30 min, slow walking speed, history of cerebrovascular disease, increased HbA1c and UA levels, increased eGFR, decreased LDL-C level, and proteinuria. For deaths from malignant neoplasm in the respiratory tract, the independent risk factors were male sex, older age, slow walking speed, smoking history, increased HbA1c level, and decreased hemoglobin level.

**Table 5:** Cox proportional hazards analysis for the independent risk factors in respiratory disease mortality.

	Hazard ratio	95% CI		p value
Respiratory diseases including malignant neoplasm (n = 1263)				
Sex (men)	3.750	3.056	4.623	< 0.0001*
Age (+ 1 year)	1.106	1.090	1.122	< 0.0001*
Body mass index (+ 1 kg/m^2^)	0.915	0.892	0.939	< 0.0001*
Systolic blood pressure (+ 1 mmHg)	0.995	0.995	1.004	0.6863
Habit of walking and/or physical activity	0.839	0.718	0.979	0.0261*
Fast walking speed	0.518	0.439	0.610	< 0.0001*
Smoking history	1.941	1.627	2.309	< 0.0001*
Drinking habits	0.617	0.523	0.729	< 0.0001*
Adequate sleep	1.170	0.965	1.430	0.1114
History of cerebrovascular diseases	1.623	1.247	2.079	0.0005*
History of cardiovascular diseases	1.273	0.991	1.613	0.0588
Hemoglobin A1c (+ 1%)	1.213	1.127	1.298	< 0.0001*
Low density lipoprotein cholesterol (+ 1 mg/dl)	0.995	0.992	0.997	< 0.0001*
Alanine aminotransferase (+ 1 U/L)	1.001	0.997	1.005	0.5408
Uric acid (+ 1 mg/dl)	1.056	1.012	1.084	0.0161*
Creatinine (+ 1 mg/dl)	1.132	0.902	1.308	0.2430
Urinary protein (positive)	1.432	1.189	1.716	0.0002*
Respiratory diseases excluding malignannt neoplasm (n = 437)				
Sex (men)	3.898	2.879	5.355	< 0.0001*
Age (+ 1 year)	1.141	1.111	1.173	< 0.0001*
Body mass index (+ 1 kg/m^2^)	0.831	0.790	0.874	< 0.0001*
Diastolic blood pressure (+ 1 mmHg)	0.989	0.977	1.001	0.0703
Weight gain more than 10 kg since age 20	1.083	0.767	1.515	0.6470
Exercise habit for more than 30 min	0.591	0.442	0.783	0.0002*
Fast walking speed	0.274	0.197	0.375	< 0.0001*
History of cerebrovascular diseases	2.049	1.379	2.950	0.0006*
History of cardiovascular diseases	1.336	0.893	1.936	0.1537
History of chronic renal failure	2.175	0.765	4.831	0.1309
Hemoglobin A1c (+ 1%)	1.241	1.108	1.366	0.0005*
Low density lipoprotein cholesterol (+ 1 mg/dl)	0.995	0.991	0.999	0.0281*
Aspartate aminotransferase (+ 1 U/L)	1.002	0.996	1.005	0.3752
Uric acid (+ 1 mg/dl)	1.066	1.000	1.101	0.0497*
Estimated glomerular filtration rate (+ 1 mL/min/1.73m^2^)	1.006	1.003	1.007	0.0003*
Urinary protein (positive)	1.876	1.396	2.491	< 0.0001*
Malignant neoplasm of respiratory tract (n = 826)				
Sex (men)	3.607	2.306	5.723	< 0.0001*
Age (+ 1 year)	1.096	1.064	1.131	< 0.0001*
Waist circumference (+ 1 cm)	1.014	0.996	1.032	0.1242
Systolic blood pressure (+ 1 mmHg)	0.999	0.989	1.008	0.7573
Fast walking speed	0.629	0.456	0.860	0.0035*
Smoking history	3.287	2.349	4.586	< 0.0001*
Drinking habits	0.842	0.601	1.187	0.3247
Adequate sleep	1.187	0.806	1.810	0.3955
History of cerebrovascular diseases	1.650	0.955	2.671	0.0711
History of cardiovascular diseases	0.717	0.375	1.244	0.2507
Hemoglobin A1c (+ 1%)	1.209	1.035	1.380	0.0188*
Low density lipoprotein cholesterol (+ 1 mg/dl)	0.996	0.991	1.002	0.1794
Alanine aminotransferase (+ 1 U/L)	1.000	0.988	1.006	0.9749
Hemoglobin (+ 1 g/dl)	0.884	0.792	0.993	0.0376*
Uric acid (+ 1 mg/dl)	1.011	0.897	1.091	0.8525
Creatinine (+ 1 mg/dl)	1.068	0.537	1.475	0.8094
Urinary protein (positive)	0.964	0.634	1.421	0.8583

Asterisk means p < 0.05, statistically significant.

## Discussion

In this study, we determined the impact of lifestyle factors and health status on deaths from respiratory diseases in the general Japanese population using data obtained from a community health examination database. We followed > 600,000 individuals for 7 years; of these,  > 8000 participants died. The analysis of the causes of deaths showed that respiratory diseases, including malignant neoplasms, accounted for 15.69% of all deaths, next to cardiovascular diseases. The significant risk factors of death from respiratory disease including malignant neoplasm were male sex, older age, lower BMI, no walking habit or physical activity, slow walking speed, history of smoking, no drinking habits, history of cerebrovascular disease, increased HbA1c and UA levels, decreased LDL-C level, and proteinuria. Particularly, in this study, we showed for the first time that exercise habits, such as walking and physical activity, as well as a fast walking speed reduced the risk of mortality from all respiratory diseases.

In previous studies using health checkup data, various backgrounds, underlying diseases, and biomarkers were found to be the risk factors for future mortality. In our previous cohort study involving a general population, male sex, older age, high d-dimer and fibrinogen levels, lower BMI and total cholesterol levels, and history of stroke and gastric ulcer were independent risk factors for respiratory death^16^. The other analyses showed that impaired pulmonary function was an independent risk factor of death from cardiac and respiratory diseases^3^. Several studies have reported that physical activity, such as walking, has been shown to reduce the risk of respiratory disease^9,10,14,17^. A higher frequency of running and walking was reported to reduce the risk of death from respiratory disease, pneumonia, and aspiration pneumonia in a dose-dependent manner^14^. Furthermore, studies on patients with COPD or lung cancer have reported beneficial effects of exercise or physical activity on the clinical course of the disease^9,17^. These results are consistent with our findings showing that physical activity is beneficial to the prognosis of respiratory diseases; however, our results also differed, as the previous studies recruited walkers or patients with specific diseases were targeted. In our previous study, our health checkup data showed that daily walking habits reduced the risk of death from pneumonia^10^. We considered that the present study is valuable, as our health checkup data showed that walking habit and physical activity or fast walking speed can reduce the risk of mortality from all respiratory diseases, including malignant neoplasm, as well as pneumonia in the future. In the present study, malignant neoplasm was the leading cause of death from respiratory disease. The risk factors of death from respiratory disease without and without malignant neoplasms in the respiratory tract were different (Table 5). The risk factors common to both respiratory diseases with and without malignant neoplasms were male sex, older age, fast walking speed, and higher HbA1c level. Some underlying diseases, such as cerebrovascular or chronic renal disease, and some laboratory data, such as LDL-C, UA, and urinary protein were significant risk factors of death from respiratory disease not including those with malignant neoplasms. The risk factors for death from malignant neoplasm in the respiratory tract were fewer than those from respiratory diseases as a whole or from respiratory diseases without malignant neoplasms. Only an increase in HbA1c level, a marker of diabetes mellitus, and a decrease in hemoglobin level were significantly associated with death from malignant neoplasm. Previous reports have shown that the presence of diabetes or anemia is associated with a poor prognosis of lung cancer^18,19^, consistent with the results of this study. Thus, estimating the risk of death from malignant neoplasm in the respiratory tract differed from that caused by other respiratory diseases.

Our study showed that, in addition to walking habits or physical activity, individuals with faster walking speed as compared to other individuals of same age and sex have decreased risk for mortality from respiratory disease. Another study investigated that the group with a reduced erector spinae muscle area measured by computed tomography showed a worse life prognosis in patients with COPD^20^. The erector spinae muscle, one of the antigravity muscles, is involved in the maintenance of normal posture. These muscles tend to show atrophy in patients with reduced activity. A previous study on patients with pneumonia reported that the erector spinae of these patients who died or required rehabilitation were significantly smaller than those who discharged^7^. Furthermore, a study on nontuberculous mycobacteria also reported that patients with reduced erector spinae muscle area had a poorer prognosis^21^. The serum level of irisin, one of the myokines secreted by the skeletal muscle cells, was associated with physical activity in patients with COPD. A lower serum irisin level was shown in patients with COPD than in controls. Moreover, all participants with a lower physical activity level had lower serum irisin levels^22^. Decreased muscle mass is associated with decreased physical activity. These results indicate that decreased muscle and muscle strength, as well as lower physical activity are strongly correlated with the prognosis of various respiratory diseases. These findings seem to support the findings of the present study, demonstrating that faster walking speed and walking habit/physical activity can reduce the risk of mortality from respiratory diseases. Pre-existing medical conditions and lifestyle habits that contribute to a decline in ADL are also risk factors, and behavioral changes and interventions that promote exercise may reduce the risk of deaths from respiratory disease.

However, the present study has some limitations. First, the studied patients were from several prefectures, which may lead to a regional bias. Second, the distribution of deaths from respiratory disease is not consistent with the Japanese mortality statistics. Third, answers to questionnaires such as smoking and drinking habits as well as walking habit or physical activity are not as objective as laboratory data. In addition, no previous information on smoking, alcohol consumption or physical activity was obtained, only current conditions were obtained. Despite these limitations, the analysis of very large scale data from > 600,000 participants in Japan can be considered a strong point of this study.

In conclusion, older age and a decline in physical activity are significant risk factors for mortality associated with respiratory diseases, regardless of smoking status. Exercise is important to reduce the risk of mortality from respiratory disease, including malignant neoplasms in the respiratory tract.

## Materials and methods

This study (J-SHC study) was conducted on general population who had undergone the nationwide screening program of the Specific Health Check-up and Guidance System (Tokutei-Kenshin), targeting the general population in Japan from 2008 to 2010. The Specific Health Check-up is an annual health checkup for all Japanese citizens aged 40–74, which has been conducted by the National Health Insurance since 2008. Our study was a prospective cohort study conducted in 82 municipalities in 7 prefectures and enrolled 664,926 people (284,320 men and 380,606 women). In this study, participants answered a self-administered questionnaire that covers their background (age, sex, body weight change), their medical histories (stroke, cardiovascular diseases, hypertension, diabetes mellitus, dyslipidemia, proteinuria, hyperuricemia) and their current lifestyles (smoking status, exercise habits, walking habits, walking speed, drinking habits and sleep condition). Individual records of participants were anonymously provided. The underlying causes of death were coded according to International Classification of Diseases (ICD)-10. Baseline information in this study included age, sex, smoking habits (ex- or non-smoker/current smoker), drinking habits (rarely, sometimes, daily) and dose, daily walking or exercise habits, dietary habits, such as frequency and duration of meals, changing of body weights, medication status for hypertension and diabetes, and dyslipidemia, medical history (previously diagnosed heart disease/stroke, chronic kidney disease, or anemia) were obtained by interviewing the examinees. For smoking habits, participants who currently smoke were defined as smokers. Past smokers were not included as smokers. For drinking habits, participants who drunk daily or sometimes were defined as habitual drinkers. For sleep, participants who had adequate sleep were defined as those who answered in the questionnaire that they were getting adequate sleep. Heights, weights, and blood pressure of participants were measured by trained staffs. Their blood pressure was measured using a standard or automatic sphygmomanometer on the right arm after 5 min of rest in a sitting position.

Habits of exercise other than walking were captured based on "yes" or "no" answers to the following question: "Do you exercise for at least 30 min at a time, twice a week, for at least 1 year, to a light sweat? Habits of daily walking were captured based on "yes" or "no" answers to the question, "In your daily life, do you walk or engage in equivalent physical activity for at least 1 h per day?" Their walking abilities were captured based on "yes" or "no" answers to the question, “Do you walk faster than your same sex of approximately the same age?”.

We took blood samples from the participants and obtained laboratory data such as their blood counts, liver function, renal function, fasting blood glucose, glycated hemoglobin A1c (HbA1c), uric acid (UA), and lipid levels. All blood analyses were performed at a local laboratory. Participants with diabetes (fasting blood glucose level of 126 or higher, glycated hemoglobin A1c level of 6.5% or higher, or use of medication for diabetes) and/or hypertension (systolic blood pressure of 140 mmHg or higher, diastolic blood pressure of 90 mmHg or higher, or use of medication for hypertension) were determined through a medical interview and objective data.

This study was conducted in accordance with the Declaration of Helsinki guidelines. This study was approved by the Ethics Committee of Yamagata University (Approval No. 2008-103). All data were anonymized before analysis; therefore, the ethics committee of Yamagata University waived the need for informed consent from study participants. As a statistical analysis of this study, we estimated hazard ratios for mortality associated with respiratory disease using a Cox regression model. Significance was inferred for p values of < 0.05. Statistical analyses were performed using JMP version 11.0 software (SAS Institute, Cary, NC, USA).

## Data Availability

The dataset used in this study is not publicly available due to a restriction by agreement among the research group members.

## References

[1] World Health Organization. Life expectancy at birth (years). https://www.who.int/data/gho/data/indicators/indicator-details/GHO/life-expectancy-at-birth-(years) (Accessed 23th October 2022).

[2] Tai SY, Cheon S, Yamaoka Y, Chien YW, Lu TH (2022). Changes in the rankings of leading causes of death in Japan, Korea, and Taiwan from 1998 to 2018: A comparison of three ranking lists. BMC Public Health.

[3] Shibata Y, Inoue S, Igarashi A, Yamauchi K, Abe S, Aida Y, Nunomiya K, Sato M, Nakano H, Sato K, Nemoto T, Kimura T, Watanabe T, Konta T, Daimon M, Ueno Y, Kato T, Kayama T, Kubota I (2013). A lower level of forced expiratory volume in 1 second is a risk factor for all-cause and cardiovascular mortality in a Japanese population: The Takahata study. PLoS One.

[4] Covinsky KE, Palmer RM, Fortinsky RH, Counsell SR, Stewart AL, Kresevic D (2003). Loss of independence in activities of daily living in older adults hospitalized with medical illnesses: Increased vulnerability with age. J. Am. Geriatr. Soc..

[5] Konomura K, Nagai H, Akazawa M (2017). Economic burden of community-acquired pneumonia among elderly patients: A Japanese perspective. Pneumonia.

[6] Girard TD, Self WH, Edwards KM, Grijalva CG, Zhu Y, Williams DJ, Jain S, James Jackson JC. (2018). Long-term cognitive impairment after hospitalization for community-acquired pneumonia: A prospective cohort study. J. Gen. Intern. Med..

[7] Minegishi Y, Inoue S, Sato K, Abe K, Murano H, Furuyama K, Yang S, Machida H, Nakano H, Sato M, Nemoto T, Sato C, Nishiwaki M, Kimura T, Yamauchi K, Igarashi A, Tokairin Y, Shibata Y, Watanabe M (2019). Smaller erector spinae muscle size is associated with inability to recover activities of daily living after pneumonia treatment. Respir. Investig..

[8] World Health Organization. Life expectancy and healthy life expectancy data by country. https://apps.who.int/gho/data/view.main.SDG2016LEXv?lang=en (Accessed 23th October 2022).

[9] Waschki B, Kirsten A, Holz O, Müller KC, Meyer T, Watz H, Magnussen H (2011). Physical activity is the strongest predictor of all-cause mortality in patients with COPD: A prospective cohort study. Chest.

[10] Ikeda T, Inoue S, Konta T, Murakami M, Fujimoto S, Iseki K, Moriyama T, Yamagata K, Tsuruya K, Narita I, Kondo M, Shibagaki Y, Kasahara M, Asahi K, Watanabe T (2020). Can daily walking alone reduce pneumonia-related mortality among older people?. Sci. Rep..

[11] Williams PT (2014). Dose-response relationship between exercise and respiratory disease mortality. Med. Sci. Sports Exerc..

[12] Inoue Y (2007). Risk and protective factors related to mortality from pneumonia among middle-aged and elderly community residents: The JACC study. J. Epidemiol..

[13] Ukawa S (2019). Associations of daily walking time with pneumonia mortality among elderly individuals with or without a medical history of myocardial infarction or stroke: Findings from the Japan Collaborative Cohort Study. J. Epidemiol..

[14] Williams PT, Thompson PD (2013). The relationship of walking intensity to total and cause-specific mortality. Results from the national walkers’ health study. PLoS One.

[15] Wakasugi M, Narita I, Iseki K, Asahi K, Yamagata K, Fujimoto S, Moriyama T, Konta T, Tsuruya K, Kasahara M, Shibagaki Y, Kondo M, Watanabe T, Japan Specific Health Checkups (J-SHC) Study Group (2022). Healthy lifestyle and incident hypertension and diabetes in participants with and without chronic kidney disease: The Japan Specific Health Checkups (J-SHC) Study. Intern. Med..

[16] Kobayashi M, Shibata Y, Inoue S, Igarashi A, Sato K, Sato M, Nemoto T, Abe Y, Nunomiya K, Nishiwaki M, Tokairin Y, Kimura T, Daimon M, Makino N, Watanabe T, Konta T, Ueno Y, Kato T, Kayama T, Kubota I (2016). Predictors for mortality from respiratory failure in a general population. Sci. Rep..

[17] Lei J, Yang J, Dong L, Xu J, Chen J, Hou X, Bai Z (2022). An exercise prescription for patients with lung cancer improves the quality of life, depression, and anxiety. Front. Public Health.

[18] Rao Kondapally Seshasai S, Kaptoge S, Thompson A, Di Angelantonio E, Gao P, Sarwar N, Whincup PH, Mukamal KJ, Gillum RF, Holme I, Njølstad I, Fletcher A, Nilsson P, Lewington S, Collins R, Gudnason V, Thompson SG, Sattar N, Selvin E, Hu FB, Danesh J, Emerging Risk Factors Collaboration (2011). Diabetes mellitus, fasting glucose, and risk of cause-specific death. N. Engl. J. Med..

[19] Liu Y, Bai YP, Zhou ZF, Jiang CR, Xu Z, Fan XX (2019). Preoperative anemia as a prognostic factor in patients with lung cancer: A systematic review and meta-analysis of epidemiological studies. J. Cancer.

[20] Tanimura K, Sato S, Fuseya Y, Hasegawa K, Uemasu K, Sato A, Oguma T, Hirai T, Mishima M, Muro S (2016). Quantitative assessment of erector spinae muscles in patients with chronic obstructive pulmonary disease. Novel chest computed tomography-derived index for prognosis. Ann. Am. Thorac. Soc..

[21] Asakura T, Yamada Y, Suzuki S, Namkoong H, Okamori S, Kusumoto T, Niijima Y, Ozaki A, Hashimoto M, Yagi K, Kamata H, Funatsu Y, Ishii M, Jinzaki M, Betsuyaku T, Hasegawa N (2018). Quantitative assessment of erector spinae muscles in patients with *Mycobacterium*
*avium* complex lung disease. Respir. Med..

[22] Ijiri N, Kanazawa H, Asai K, Watanabe T, Hirata K (2015). Irisin, a newly discovered myokine, is a novel biomarker associated with physical activity in patients with chronic obstructive pulmonary disease. Respirology.

